# Combined utility of white blood cell count and blood glucose for predicting in-hospital outcomes in acute ischemic stroke

**DOI:** 10.1186/s12974-019-1422-7

**Published:** 2019-02-14

**Authors:** Shoujiang You, Zhijie Ou, Wei Zhang, Danni Zheng, Chongke Zhong, Xiaofeng Dong, Chenhong Qiu, Taosheng Lu, Yongjun Cao, Chun-Feng Liu

**Affiliations:** 1 Department of Neurology and Suzhou Clinical Research Center of Neurological Disease, The Second Affiliated Hospital of Soochow University, No.1055, Sanxiang Road, Suzhou, 215004 Jiangsu China; 2 Department of Neurology, Changshu TCM Hospital Affiliated to Nanjing University of Chinese Medicine, Suzhou, 215000 China; 3 Centre for Big Data Research in Health, University of New South Wales, Sydney, NSW Australia; 4 The George Institute for Global Health, Faculty of Medicine, University of New South Wales, Sydney, NSW Australia; 5 Department of Epidemiology, School of Public Health, Medical College of Soochow University, Suzhou, 215123 China; 6 Department of Neurology, Suzhou Hospital Affiliated to Nanjing Medical University, Suzhou, 215001 China; 7 Department of Neurology, Changshu First People’s Hospital, Suzhou, 215500 China; 8 Institutes of Neuroscience, Soochow University, Suzhou, 215123 China

**Keywords:** Acute ischemic stroke, White blood cell, Blood glucose, Combined effect, In-hospital outcomes

## Abstract

**Background:**

High white blood cell (WBC) count and high blood glucose level are risk factors for mortality and pneumonia after acute ischemic stroke (AIS). We investigated the combined effect of high WBC count and high blood glucose level on hospital admission and in-hospital mortality and pneumonia in acute AIS patients.

**Methods:**

A total of 3124 AIS patients enrolled from December 2013 to May 2014 across 22 hospitals in Suzhou city were included in the present study. We divided patients into four groups according to their level of WBC count and blood glucose: NWNG (normal WBC count and normal glucose), NWHG (normal WBC count and higher glucose), HWNG (higher WBC count and normal glucose), and HWHG (higher WBC count and higher glucose). Cox proportional hazard model and logistic regression model were used to estimate the combined effect of WBC count and blood glucose on all-cause in-hospital mortality and pneumonia in AIS patients.

**Results:**

HWHG was associated with a 2.22-fold increase in the risk of in-hospital mortality in comparison to NWNG (adjusted hazard ratio [HR] 2.22; 95% confidence interval [CI], 1.21–4.07; *P* trend = 0.003). The risk of pneumonia was significantly higher in patients with HWHG compared to those with NWNG (adjusted odds ratio [OR] 2.61; 95% CI, 1.66–4.10; *P* trend < 0.001). The C-statistic for the combined WBC count and blood glucose was higher than WBC count or blood glucose alone for prediction of in-hospital mortality and pneumonia (all *p* < 0.01).

**Conclusions:**

High WBC count combined with high blood glucose level at admission was independently associated with in-hospital mortality and pneumonia in AIS patients. Moreover, the combination of WBC count and blood glucose level appeared to be a better predictor than WBC count or blood glucose alone.

**Electronic supplementary material:**

The online version of this article (10.1186/s12974-019-1422-7) contains supplementary material, which is available to authorized users.

## Introduction

Increased white blood cell (WBC) count and high glucose level are frequently found in patients with acute ischemic stroke (AIS) [1–4]. Several studies indicated that high WBC and high glucose level were not only associated with high risk of poor outcome and mortality but also pneumonia after stroke [2, 4–10].

Stress and inflammatory response are the two main causative factors leading to higher WBC and high glucose associated with mortality and pneumonia after acute stroke [2, 10–13]. There may exist a combined effect of WBC count and blood glucose level on stroke outcomes based on the similar mechanisms. Studies found that acute myocardial infarction (AMI) patients with co-existing higher WBC and high blood glucose had higher rates of in-hospital mortality and poor outcome compared to those with higher WBC or high blood glucose alone [14, 15]. A US study of 436 ischemic stroke patients indicated that those with both elevated total WBC count and high blood glucose had poor discharge outcome independent of elevations in either factor alone [16]. However, evidence on the association between the combined effect of WBC count and blood glucose at admission and in-hospital mortality and pneumonia in AIS patients are limited.

In the present study, we aimed to evaluate the possible association between combined effect of WBC count and blood glucose and in-hospital mortality and pneumonia in a large multicenter study of over 3000 AIS patients from Suzhou, China.

## Methods

### Study participants

**Fig. 1:**
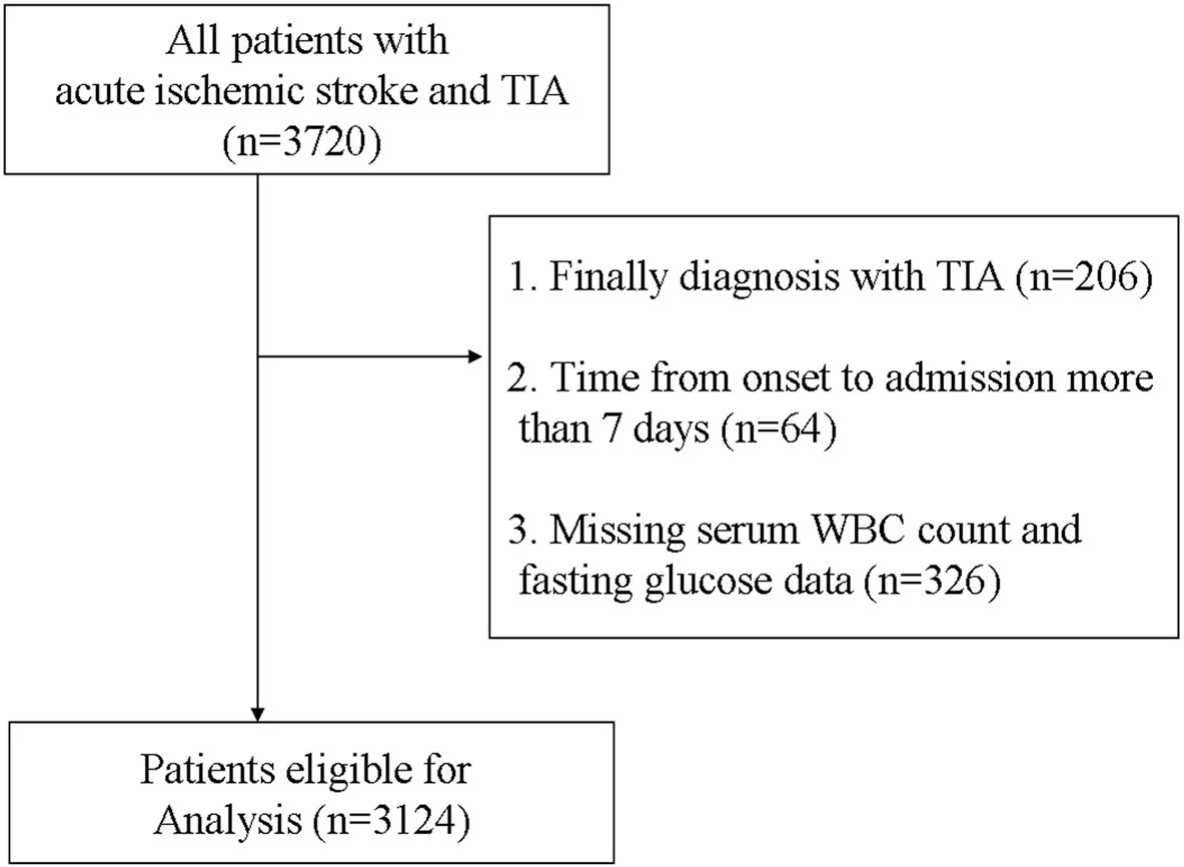
Patient flowchart. TIA indicates transient ischemic attack. WBC, white blood cell

From December 2013 to May 2014, we recruited patients with AIS or transient ischemic attack (TIA) from 22 hospitals in Suzhou, China (Additional file 1). Patients aged ≥ 18 years with a clinical diagnosis of AIS or TIA were considered eligible. Diagnosis of ischemic stroke was made according to the World Health Organization-defined criteria based on patient history, clinical data, and neuroimaging results (computed tomography [CT] or magnetic resonance imaging [MRI]). A team of investigators, including neurologists, reviewed the eligibility of study participants. Additional exclusion criteria were as follows: (1) diagnosis of TIA based on completely revised of symptoms and no acute infarct on the MRI or follow-up CT scans, (2) time from onset to admission over 7 days, and (3) lack of data on admission WBC or fasting glucose levels. Three thousand one hundred twenty-four patients were potentially eligible for this analysis (flowchart of participants selection; Fig. 1).

### Data collection and outcome assessment

We collected baseline information, including patient demographics, vascular risk factors, stroke severity (as measured by the National Institutes of Health Stroke Scale, NIHSS, and modified Rankin Scale, mRS), medication use, imaging data, and diagnosis-related information. Vascular risk factors included history of stroke, history of hypertension, history of diabetes mellitus, history of atrial fibrillation, history of coronary heart disease, current or previous smoking status, and alcohol consumption. Information on the aforementioned factors were obtained by interviews with patients or their family members (if patients were not able to communicate). Current smoking status was defined as having smoked at least one cigarette per day for the previous year or more. Data on the amount and type of alcohol consumed during the past year was collected. Alcohol consumption was defined as having consumed at least one alcoholic drink per day during the last year. Hypertension was defined as having a systolic blood pressure (BP) ≥ 140 mmHg and/or diastolic BP ≥ 90 mmHg or use of antihypertensive medications. Diabetes mellitus was defined as having fasting glucose ≥ 7.0 mmol/L (126 mg/dL), non-fasting glucose ≥ 11.1 mmol/L (200 mg/dL) with classic symptoms of hyperglycemia or hyperglycemic crisis, and use of glucose-lowering drugs. Atrial fibrillation was defined as having a history of atrial fibrillation, confirmed by ≥ 1 electrocardiogram or the presence of arrhythmia during hospitalization. Pneumonia after AIS was diagnosed by treating physicians according to the criteria of US Center for Disease Control and Prevention for hospital-acquired pneumonia, based on clinical and laboratory test [17]. Blood samples were collected within 24 h of hospital admission. The WBC count was determined at hospital admission by automated cell counters via standard techniques, and blood glucose and other biochemical parameters were analyzed enzymatically by automatic biochemical analyzer using fasting blood at local laboratories. The outcome of this study was all-cause in-hospital mortality and pneumonia.

### Statistical analysis

Study participants were divided into four groups, based on serum WBC count and fasting glucose levels at admission: normal WBC count and normal glucose, NWNG (WBC count < 10 × 10^9^/L and fasting glucose < 7.0 mmol/L), normal WBC count and higher glucose, NWHG (WBC count < 10 × 10^9^/L and fasting glucose ≥ 7.0 mmol/L), higher WBC count and normal glucose, HWNG (WBC count ≥ 10 × 10^9^/L and fasting glucose < 7.0 mmol/L), and higher WBC count and higher glucose, HWHG (WBC count ≥ 10 × 10^9^/L and fasting glucose ≥ 7.0 mmol/L) [18, 19]. Continuous variables were expressed as mean ± standard deviation (SD) or median (interquartile range [IQR]) and were compared using the analysis of variance or Wilcoxon rank-sum test. Categorical variables were expressed as frequency (%) and were compared using the chi-square test.

The crude cumulative risks of in-hospital mortality for each patient group based on admission serum WBC count and blood glucose were shown in a Kaplan-Meier plot and compared using the log-rank test. Crude and multivariable Cox proportional hazards regression model and logistic regression models were used to estimate the risk of in-hospital mortality and the risk of in-hospital pneumonia respectively. Hazard ratios (HRs), odds ratios (ORs), and 95% confidence interval (CIs) were calculated for each group with the NWNG as reference. Potential confounders that were adjusted in the multivariable models included age, sex, systolic BP, body temperature, time from onset to admission, cigarette smoking status, alcohol drinking, history of hypertension, history of diabetes mellitus, history of coronary heart disease, history of atrial fibrillation, history of stroke, thrombolysis treatment, baseline NIHSS score, Oxfordshire Community Stroke Project (OCSP) classification, and estimated glomerular filtration rate (eGFR) levels. To assess the robustness of the association between different serum WBC count and blood glucose levels and in-hospital mortality and pneumonia, we also performed sensitivity analyses (by restricting to patients with first-ever strokes and those with time from onset to admission ≤ 24 h) and conducted subgroup analysis (in patients with or without diabetes). In addition, we tested the discriminatory ability of WBC count and blood glucose (combined and separately) to predict in-hospital mortality and pneumonia by calculating C-statistics (areas under receiver operating characteristic [ROC] curves). All *P* values were two-tailed, and a significance level of 0.05 was used. All analyses were conducted using the SPSS Version 17.0 statistical software.

## Results

Complete data on conventional risk factors and WBC count and blood glucose levels at admission were available for 3124 patients whose mean age was 68.6 years (± 12.9), with a median NIHSS score of 4.0 (IQR, 2.0–7.0). In comparison to NWNG participants, those with HWHG were more likely to be younger, male, and had more severe stroke (higher NIHSS) and other co-morbidities including hypertension, diabetes mellitus, coronary heart disease, and atrial fibrillation. HWHG patients also differed in metabolic profile (higher fasting glucose levels and serum total cholesterol, low-density lipoprotein cholesterol and WBC count level, and higher baseline diastolic BP and shorter time from onset to hospital) (Table 1).

**Table 1:** Baseline characteristics of 3124 acute ischemic stroke patients according to white blood cell and blood glucose level

Characteristics^a^	NWNG	NWHG	HWNG	HWHG	*P* value
Number of subjects	2025	681	266	152	
Demographics					
Age, years	68.8 ± 12.8	68.8 ± 11.4	66.5 ± 16.3	67.6 ± 14.3	0.031
Male sex	1181 (58.3)	356 (52.3)	170 (63.9)	93 (61.2)	0.004
Cigarette smoking status	420 (20.7)	119 (17.5)	54 (20.3)	25 (16.4)	0.206
Alcohol consumption	205 (10.1)	62 (9.1)	21 (7.9)	16 (10.5)	0.617
Clinical features					
Time from onset to hospital, hours	24.0 (6.0–72.0)	24.0 (5.0–72.0)	24.0 (4.0–48.0)	12.0 (3.0–48.0)	< 0.001
Hospital stay, days	10.0 (8.0–13.0)	11.0 (8.0–15.0)	11.0 (7.0–15.0)	12.0 (7.5–19.0)	< 0.001
Baseline systolic BP, mm Hg	151.0 ± 22.6	154.6 ± 22.0	153.4 ± 25.0	155.5 ± 22.6	0.001
Baseline diastolic BP, mm Hg	85.1 ± 13.0	85.7 ± 12.5	87.1 ± 14.7	86.1 ± 14.6	0.087
TG, mmol/L	1.2 (0.9–1.6)	1.4 (1.0–2.2)	1.1 (0.9–1.6)	1.2 (0.9–1.8)	< 0.001
TC, mmol/L	4.5 (3.8–5.1)	4.7 (4.0–5.5)	4.6 (3.9–5.3)	4.9 (4.2–5.6)	< 0.001
LDL-C, mmol/L	2.6 (2.1–3.2)	2.7 (2.1–3.4)	2.7 (2.2–3.2)	3.1 (2.3–3.7)	< 0.001
HDL-C, mmol/L	1.2 (1.0–1.4)	1.2 (1.0–1.4)	1.2 (1.0–1.4)	1.2 (1.0–1.5)	0.079
FG, mmol/L	5.3 (4.9–5.9)	8.9 (7.7–11.3)	5.6 (5.0–6.1)	9.2 (8.1–11.6)	< 0.001
WBC, 10^3^/uL	6.3 (5.2–7.5)	6.8 (5.6–8.0)	11.5 (10.6–13.0)	11.6 (10.6–13.7)	< 0.001
eGFR, ml/min/1.73 m^2^	94.3 (76.2–114.8)	99.3 (78.2–124.6)	93.2 (71.2–117.2)	94.9 (64.3–115.8)	0.004
Baseline NIHSS score	3.0 (2.0–6.0)	4.0 (2.0–7.0)	6.0 (3.0–12.0)	7.5 (3.5–15.0)	< 0.001
Medical history					
History of hypertension	1542 (76.1)	569 (83.6)	211 (79.3)	129 (84.9)	< 0.001
History of diabetes mellitus	236 (11.7)	458 (67.3)	26 (9.8)	84 (55.3)	< 0.001
History of coronary heart disease	97 (4.8)	48 (7.0)	15 (5.6)	14 (9.2)	0.029
History of atrial fibrillation	283 (14.0)	108 (15.9)	56 (21.1)	30 (19.7)	0.007
History of stroke	464 (22.9)	144 (21.1)	46 (17.3)	39 (25.7)	0.122
Medication history					
Antihypertensive therapy	1133 (56.0)	449 (65.9)	148 (55.6)	102 (67.1)	< 0.001
Antiplatelet therapy	161 (8.0)	52 (7.6)	11 (4.1)	10 (6.6)	0.162
Anticoagulation therapy	21 (1.0)	8 (1.2)	3 (1.1)	3 (2.0)	0.810
Antiglycemic therapy	179 (8.8)	338 (49.6)	19 (7.1)	49 (32.2)	< 0.001
Statin therapy	76 (3.8)	13 (1.9)	3 (1.1)	3 (2.0)	0.016
Thrombolysis treatment	43 (2.1)	11 (1.6)	12 (4.5)	11 (7.2)	< 0.001
Stroke syndrome					< 0.001
TACS	143 (7.1)	62 (9.1)	46 (17.3)	44 (28.9)	
PACS	1076 (53.1)	311 (45.7)	129 (48.5)	48 (31.6)	
POCS	416 (20.5)	209 (30.7)	70 (26.3)	48 (31.6)	
LACS	390 (19.3)	99 (14.5)	21 (7.9)	12 (7.9)	

^a^Continuous variables are expressed as mean ± standard deviation or as median (interquartile range). Categorical variables are expressed as frequency (percent)

*Abbreviations*: *BP* blood pressure, *TG* triglycerides, *TC* total cholesterol, *LDL-C* low-density lipoprotein cholesterol, *HDL-C* high-density lipoprotein cholesterol, *FG* fasting glucose, *eGFR* estimated glomerular filtration rate, *mRS* modified Rankin Scale, *NIHSS* National Institutes of Health Stroke Scale, *TACS* total anterior circulation syndrome, *PACS* partial anterior circulation syndrome, *POCS* posterior circulation syndrome, *LACS* lacunar syndrome, *Q* quartile

**Fig. 2:**
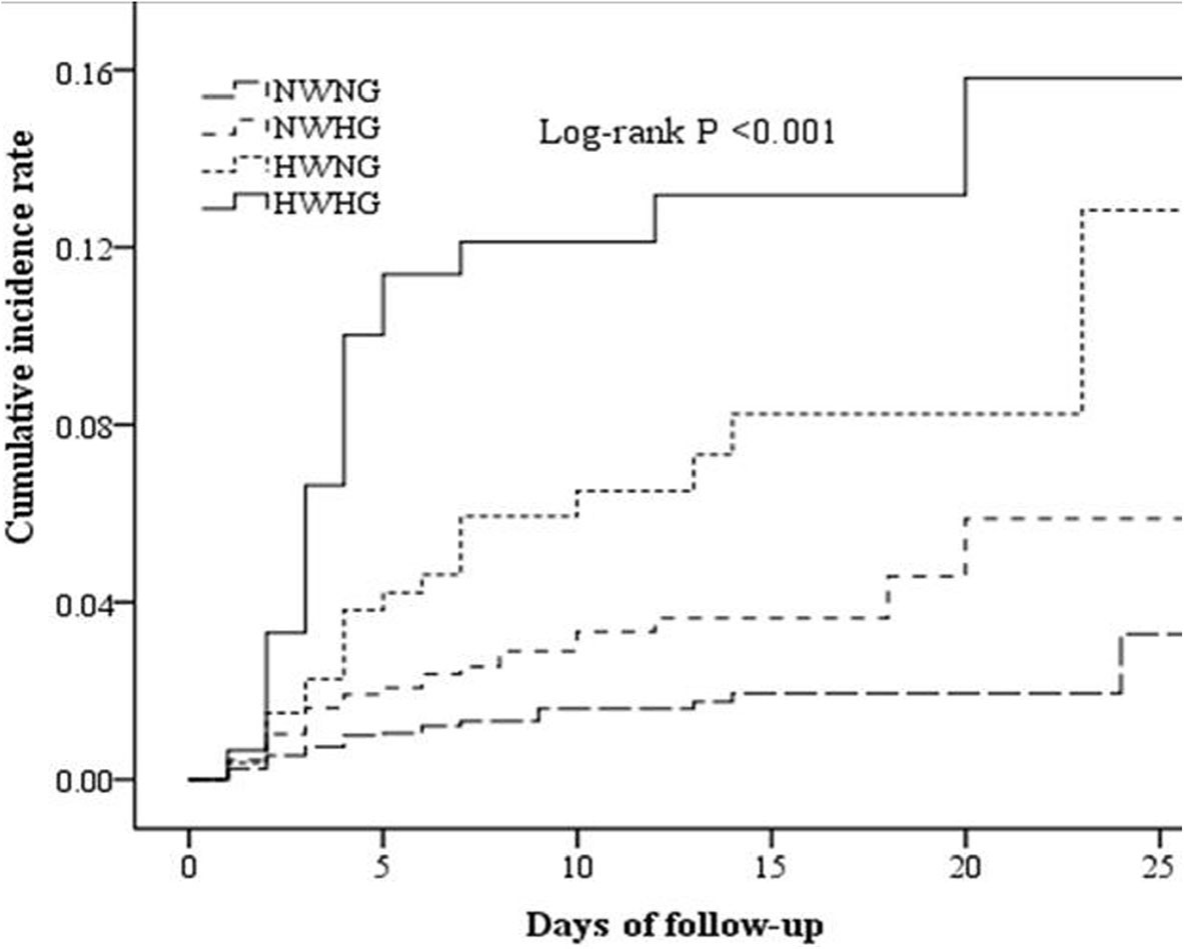
Cumulative incidence curves of in-hospital mortality by WBC count and blood glucose level. WBC indicates white blood cell; NWNG, normal WBC count and normal glucose; NWHG, normal WBC count and higher glucose; HWNG, higher WBC count and normal glucose; HWHG, higher WBC count and higher glucose

During hospitalization, 104 patients (3.3%) died from all causes. HWHG patients had the highest cumulative incidence of in-hospital mortality (log-rank *P* < 0.001; Fig. 2). In the unadjusted model, the HR of in-hospital mortality was significantly higher among study participants with admission HWHG, NWHG, and HWNG compared with NWNG (*P* trend < 0.001). After adjusting for age, sex, time from onset to admission, baseline NIHSS score, and other covariates, the HR (95% CI) of admission HWHG was 2.22 (1.21–4.07) and HWNG was 2.08 (1.15–3.78) for mortality, as compared with NWNG (*P* trend = 0.003) (Table 2). HWHG was also shown to be associated with a higher risk of in-hospital mortality in all sensitivity analyses (Table 2).

**Table 2:** Hazard ratios and 95% confidence intervals of in-hospital mortality according to level of white blood cell and blood glucose

	NWNG	NWHG	HWNG	HWHG	*P* trend
No.	2025	681	266	152	
No. of deaths	35 (1.7)	26 (3.8)	19 (7.1)	24 (15.8)	
Crude	1.00	2.02 (1.21–3.56)	3.89 (2.21–6.77)	7.59 (4.47–12.88)	< 0.001
Model 1	1.00	2.12 (1.27–3.54)	3.95 (2.26–6.91)	7.68 (4.53–13.01)	< 0.001
Model 2	1.00	1.46 (0.84–2.56)	2.08 (1.15–3.78)	2.22 (1.21–4.07)	0.003
Sensitivity analysis					
Model 3	1.00	1.28 (0.68–2.40)	1.69 (0.88–3.25)	2.11 (1.04–4.31)	0.022
Model 4	1.00	1.37 (0.75–2.50)	2.16 (1.18–3.98)	1.72 (0.88–3.37)	0.025

Model 1, adjusted for age and sex;

Model 2, adjusted for age, sex, systolic BP, body temperature, time from onset to admission, cigarette smoking status, alcohol drinking, history of hypertension, history of diabetes mellitus, history of coronary heart disease, history of atrial fibrillation, history of stroke, thrombolysis treatment, baseline National Institutes of Health Stroke Scale score, eGFR levels, Oxfordshire Community Stroke Project classification, and pneumonia

Model 3, adjusted for model 2 and further restricted to patients with first-ever stroke

Model 4, adjusted for model 2 and further restricted to patients with time from onset to admission ≤ 24 h

**Fig. 3:**
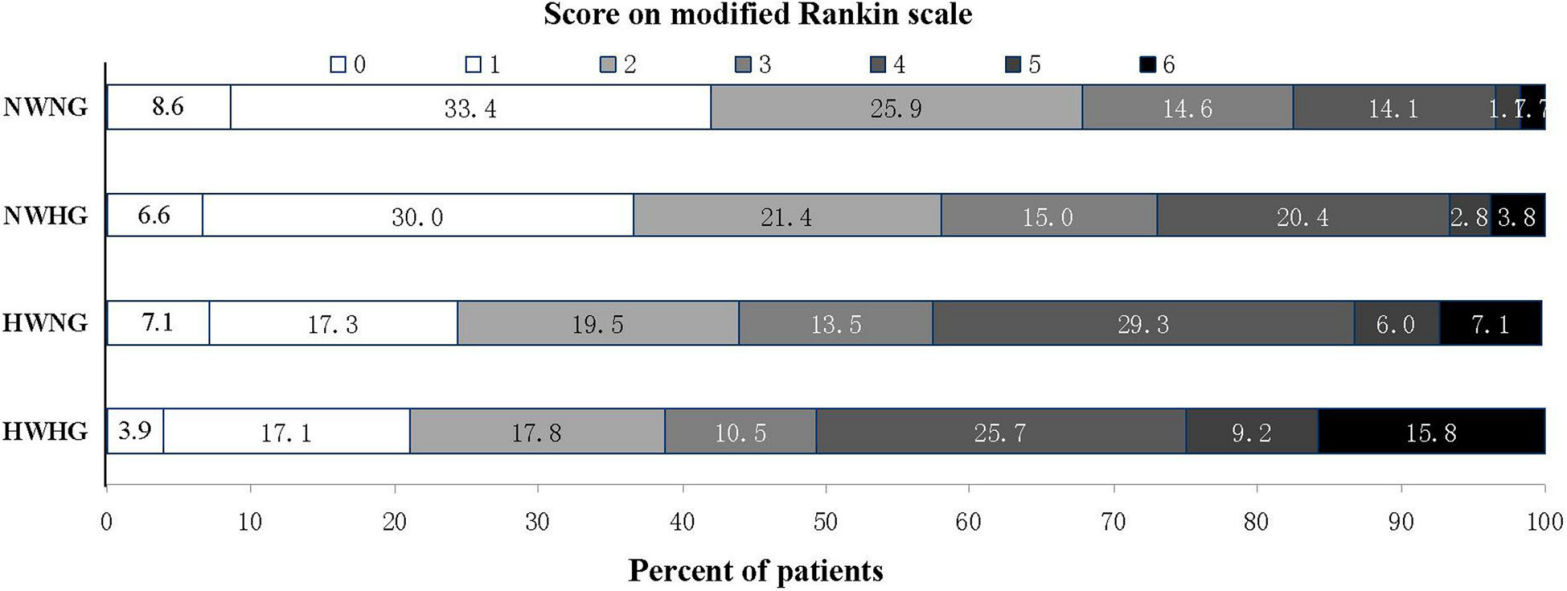
Relationship between mRS and combined WBC count and blood glucose type during hospital discharge. mRS indicates modified Rankin Scale; WBC, white blood cell; NWNG, normal WBC count and normal glucose; NWHG, normal WBC count and higher glucose; HWNG, higher WBC count and normal glucose; HWHG, higher WBC count and higher glucose

NWNG patients had a median mRS score of 2 (IQR 1–3), in comparison to HWHG patients with a median mRS score of 4 (IQR 2–4.5) during hospital discharge. Figure 3 shows the distribution of disability scores on the mRS during hospital discharge for patients classified according to combined WBC count and blood glucose levels (*p* < 0.001).

During hospitalization, 535 patients (17.1%) had pneumonia. In the unadjusted regression model, the odds of pneumonia were significantly higher among HWHG, NWHG, and HWNG participants compared with NWNG patients (*P* trend < 0.001). After adjusting for age, sex, time from onset to admission, baseline NIHSS score, eGFR, and other traditional risk factors, the OR (95% CI) for the HWHG group was 2.61 (95% CI 1.66–4.10) and HWNG group was 2.05 (95% CI 1.45–2.91) as compared with the NWNG for pneumonia (*P* trend < 0.001) (Table 3). Similar associations between HWHG, HWNG, and pneumonia were shown in all sensitivity analyses (Table 3).

**Table 3:** Odds ratios and 95% confidence intervals of pneumonia according to level of white blood cell and blood glucose

	NWNG	NWHG	HWNG	HWHG	*P* trend
No.	2025	681	266	152	
No. of Pneumonia	265 (13.1)	125 (18.4)	85 (32.0)	60 (39.5)	
Crude	1.00	1.49 (1.18–1.89)	3.12 (2.34–4.16)	4.33 (3.05–6.15)	< 0.001
Model 1	1.00	1.59 (1.25–2.02)	3.67 (2.70–5.00)	5.26 (3.61–7.66)	< 0.001
Model 2	1.00	1.34 (0.99–1.83)	2.05 (1.45–2.91)	2.61 (1.66–4.10)	< 0.001
Sensitivity analysis					
Model 3	1.00	1.13 (0.79–1.63)	2.15 (1.45–3.17)	2.43 (1.44–4.10)	< 0.001
Model 4	1.00	1.27 (0.88–1.82)	1.61 (1.06–2.45)	2.48 (1.46–4.20)	< 0.001

Model 1, adjusted for age and sex

Model 2, adjusted for age, sex, systolic BP, body temperature, time from onset to admission, cigarette smoking status, alcohol drinking, history of hypertension, history of diabetes mellitus, history of coronary heart disease, history of atrial fibrillation, history of stroke, thrombolysis treatment, baseline National Institutes of Health Stroke Scale score, Oxfordshire Community Stroke Project classification, and eGFR levels

Model 3, adjusted for model 2 and further restricted to patients with first-ever stroke

Model 4, adjusted for model 2 and further restricted to patients with time from onset to admission ≤24 h

**Fig. 4:**
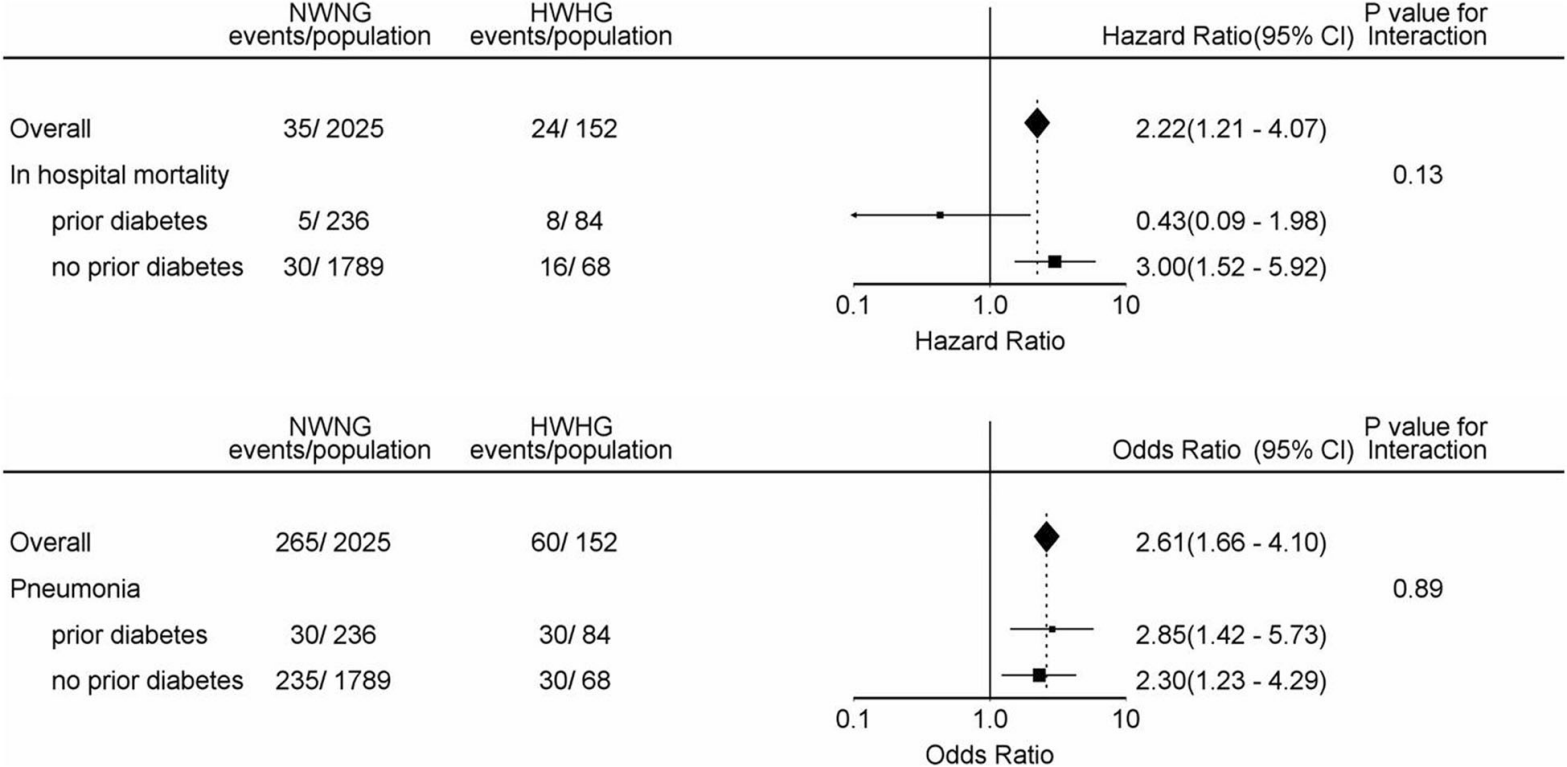
Association between co-existing higher WBC count and higher blood glucose on in-hospital mortality and pneumonia in AIS patients with or without diabetes. WBC indicates white blood cell; AIS, acute ischemic stroke; NWNG, normal WBC count and normal glucose; HWHG, higher WBC count and higher glucose

Subgroup analysis showed that HWHG was associated with in-hospital mortality and pneumonia in patients with and without diabetes. No significant interaction was observed (*P*-interaction > 0.05 for all, Fig. 4).

**Fig. 5:**
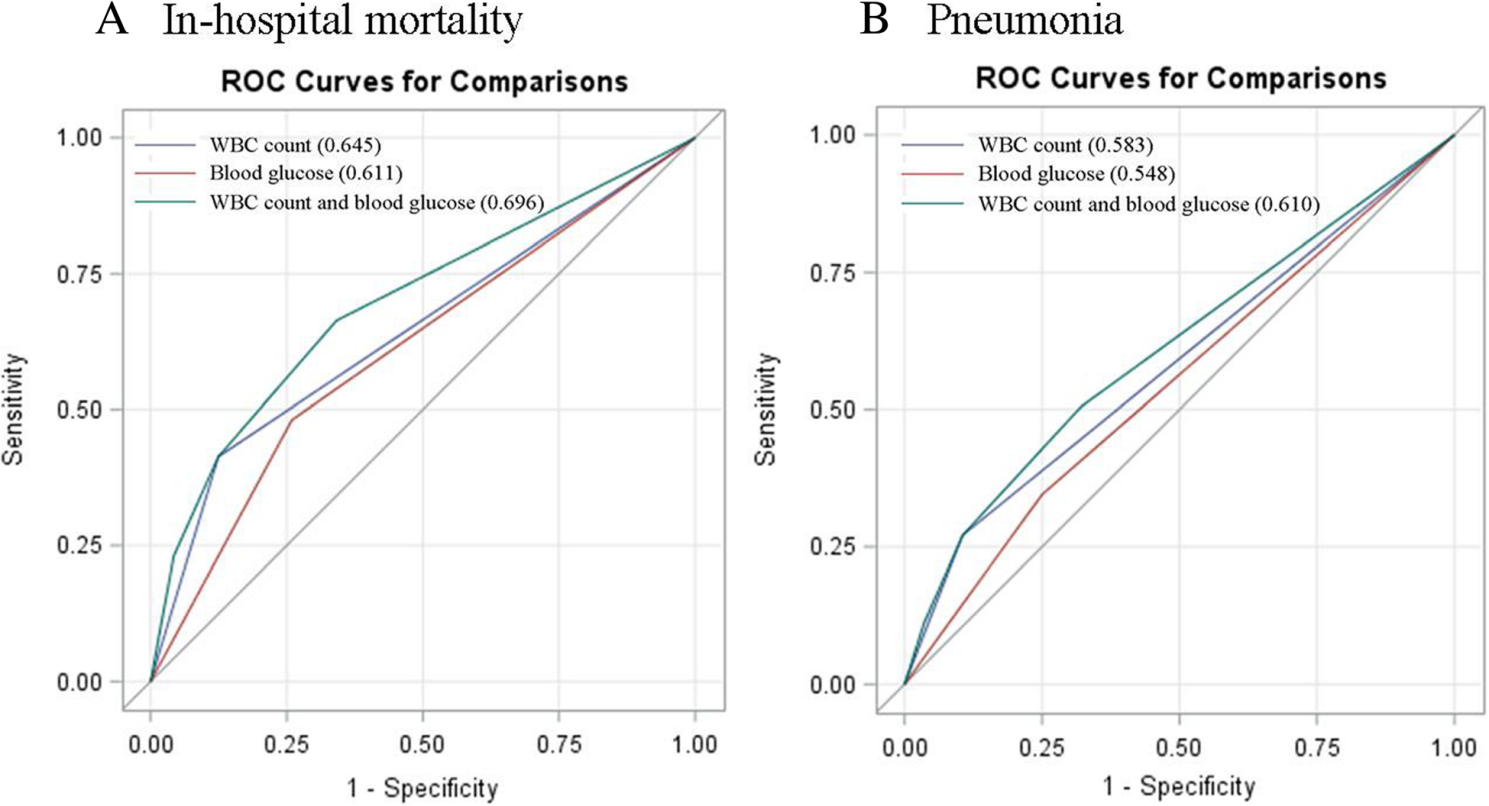
**a** ROC curve of combined WBC count and blood glucose level on in-hospital mortality. ROC indicates receiver operating characteristic; WBC, white blood cell. **b** ROC curves of combined WBC count and blood glucose level on in-hospital pneumonia. ROC indicates receiver operating characteristic; WBC, white blood cell

ROC curves comparing the predictive ability of combined WBC count and blood glucose, WBC count or blood glucose alone on in-hospital mortality and pneumonia are shown in Fig. 5a and b. The C-statistic was significantly greater for the combined effect of WBC count and blood glucose than WBC count or blood glucose alone for both outcomes (all *p* < 0.01).

## Discussion

The present study of 3124 patients demonstrated the prognostic effect of combined high WBC count and high glucose on in-hospital mortality and pneumonia in AIS. Patients with high WBC count and high glucose level appeared to be associated with a 2.22-fold and 2.61-fold increase in the risk of in-hospital mortality and pneumonia respectively, as well as poor functional outcome at discharge. The combined effect of high WBC count and high glucose on in-hospital mortality and pneumonia were comparable in patients with or without diabetes. Furthermore, the predictive value of combined WBC and glucose levels on in-hospital mortality and pneumonia appears to be better than WBC or glucose alone.

A growing body of studies has identified an association between high WBC or high glucose at admission and mortality or poor outcome after AIS [2, 4, 5, 20, 21]. However, few studies have investigated the combined effect of high WBC count and high blood glucose on poor outcome and mortality in patients with MI and AIS [14–16, 22]. It is plausible that inflammatory and stress response are the potential causative factors leading to raised WBC and blood glucose levels after AIS [2, 10–13, 23]. The Japanese Acute Coronary Syndrome Study (JACSS) reported that combined WBC count and blood glucose level were independently associated with in-hospital mortality in patients with AMI [14]. A study of 436 AIS patients also indicated that HWHG significantly increased the risk of poor outcome at hospital discharge compared to patients with NWNG [16]. Zhou et al. study also found a combined effect of hyperglycemia and inflammation markers, including elevated WBC count on the neurologic deficiency or death at discharge in AIS patients [23]. Consistent with prior literatures, the current study of 3124 AIS patients showed coexistence of high WBC count and blood glucose was associated with 2.22-fold of in-hospital mortality compared to those with normal WBC count and blood glucose. We also found the predictive value of combined WBC count and blood glucose for in-hospital mortality was better than WBC count or blood glucose alone.

Prior studies had demonstrated high WBC and high blood glucose separately as independent predictors of pneumonia after AIS [2, 6, 7, 10]. The latest study from the Netherlands also revealed that AIS patients with admission blood glucose ≥ 7.8 mmol/L had 2.31-fold increased risk of pneumonia [10]. However, little evidence exists to support an association between a combination effect of WBC count and blood glucose and the pneumonia after AIS. Our study is the first to show a combined predictive effect of WBC count and blood glucose on pneumonia after AIS and that the predictor value of combined WBC count and blood glucose is better than WBC count or blood glucose alone. These results indirectly suggest that elevated blood glucose is an inflammation marker after AIS, consistent with previous studies [10, 11, 24, 25].

Several studies including a systematic review indicated an association between high blood glucose and short-term mortality after AIS in patients without diabetes but not in diabetic patients [4, 26–28]. The relationship between admission hyperglycemia and the post-stroke pneumonia was also shown in nondiabetic patients but not in diabetic patients [10]. In the present study, we found the combined effect of WBC count and blood glucose on in-hospital mortality and pneumonia was not significantly different in patients with or without diabetes. Therefore, combined WBC count and blood glucose maybe was a more useful predictor for mortality and pneumonia for all patients (diabetic and non-diabetic).

Strengths of our study include having a large dataset of patients from multiple centers and being the first study to evaluate the combination effect of WBC count and blood glucose on in-hospital mortality and pneumonia. However, there are still some potential limitations that merit consideration. First, this cohort included some patients whose time from onset to admission exceeded 24 h; therefore, the levels of WBC and blood glucose might not accurately reflect the levels at stroke onset. However, our sensitivity analysis showed that the significance of the association remained when we restricted to patients with time from onset to admission ≤ 24 h. Secondly, a proportion of patients were excluded due to a lack of WBC and blood glucose data, which may cause selection bias. Thirdly, we were precluded investigating the possible mechanism between WBC count and blood glucose and in-hospital mortality as we lacked information on the exact cause of death. Also, data on pre-stroke disability and Trial of Org 10172 in Acute Stroke Treatment (TOAST) classification, which may also influence in-hospital mortality, were not collected. Finally, the follow-up period of our study is relatively short; thus, we were unable to evaluate the combined long-term effect of WBC count and blood glucose on AIS outcomes and the values of area under ROC curve in both models in outcome prediction were relatively low.

## Conclusion

Co-existing high WBC count and high glucose at admission was independently associated with in-hospital mortality and pneumonia in acute stroke patients and its predictive value is better than WBC count and blood glucose alone. The association between combined WBC count and glucose and in-hospital mortality and pneumonia was not different by diabetes status.

## Additional file

Additional file 1: Investigation group. (DOC 31 kb)

## Abbreviations

AIS: Acute ischemic stroke
AMI: Acute myocardial infarction
BP: Blood pressure
CI: Confidence interval
CT: Computed tomography
eGFR: Estimated glomerular filtration rate
FG: Fasting glucose
HR: Hazard ratio
HWHG: Higher WBC count and higher glucose
HWNG: Higher WBC count and normal glucose
IQR: Interquartile range
JACSS: Japanese Acute Coronary Syndrome Study
MRI: Magnetic resonance imaging
mRS: Modified Rankin Scale score
NIHSS: National Institutes of Health Stroke Scale
NWHG: Normal WBC count and higher glucose
NWNG: Normal WBC count and normal glucose
OCSP: Oxfordshire Community Stroke Project
OR: Odds ratio
ROC: Receiver operating characteristic
SD: Standard deviation
TIA: Transient ischemic attack
TOAST: Trial of Org 10172 in Acute Stroke Treatment
WBC: White blood cell
