# Supplementary material for: Combined utility of white blood cell count and blood glucose for predicting in-hospital outcomes in acute ischemic stroke
Source: J Neuroinflammation. 2019 Feb 14;16:37. doi: 10.1186/s12974-019-1422-7 (PMC6375165; doi:10.1186/s12974-019-1422-7)
Supplement: Supplementary file 1 — Investigation group. (DOC 31 kb) [file 12974_2019_1422_MOESM1_ESM.doc]

**ONLINE SUPPLEMENT**

**Combined utility of white blood cell count and blood glucose for**

**predicting in-hospital outcomes in acute ischemic stroke**

**Investigation group**

1.Department of Neurology, the Second Affiliated Hospital of Soochow University, Suzhou China(Shoujiang You, Yu Zhang, Huihui Liu, Xia Zhang, Jijun Shi, Yanlin Zhang, Zhichao Huang, Jiaping Xu, Yongjun Cao, Chunfeng Liu);

2.Department of Epidemiology, School of Public Health, Medical College of Soochow University, Suzhou, China(Chongke Zhong, Hao Peng, Yonghong Zhang);

3.The George Institute for Global Health, Royal Prince Alfred Hospital, Sydney, NSW, Australia(Danni Zheng, Xia Wang)

4.Department of Neurology, the First Affiliated Hospital of Soochow University, Suzhou, China(Jing Wang, Hongru Zhao, Xiaowei Hu, Wanli Dong, Qi Fang);

5.Department of Neurology, Suzhou Hospital Affiliated to Nanjing Medical University, Suzhou, China(Xiaofeng Dong, Huan Ye, Ling Jiang, Chenhong Qiu, Shaofang Pei, Qingzhang Cheng, Zhong Zhao, Yi Liu);

6.Department of Neurology, Suzhou Kowloon Hospital Shanghai Jiao Tong University School of Medicine, Suzhou, China(Liangfeng Fan, Shenghui Chen, Wen Li);

7.Department of Neurology, the Suzhou Hospital of Traditional Chinese Medicine, Suzhou, China(Yan Li, Qiao Han, Hongli Dong, Jin Xu);

8.Department of Neurology, The Affiliated Wujiang Hospital of Nantong University, Suzhou, China(Huaping Du, Guojie Zhai, Zhengming She, Yuan Xu);

9.Department of Neurology, Changshu First People’S Hospital, Suzhou, China(Taosheng Lu, Jiansheng Wang, Guojun Wang);

10.Department of Neurology, Changshu Second People’S Hospital, Suzhou, China(Weiting Tang, Nianxing You);

11.Department of Neurology, Zhangjiagang First People’S Hospital, Zhangjiagang, Suzhou, China(Lidan Cao, Zhou Xu, Qiuyi Wu);

12.Department of Neurology, Kunshan First People’S Hospital, Suzhou, China(Zhaoxi Ma, Qing Tang, Hongzhou Wang, Yan Zhang, Wanhua Wang);

13.Department of Neurology, Taicang First People’S Hospital, Suzhou, China(Xianhui Wang, Yijie Wu, Liyan Song, Wanqing Zhai);

14.Department of Neurology, Suzhou Xiangcheng People’S Hospital, Suzhou,215000, China(Cheng Zheng, Yongan Li);

15.Department of Neurology, Suzhou Traditional and Western Medicine hospital, Suzhou, China(Yi Luo);

16.Department of Neurology Second Affiliated Hospital of Soochow University, New District Hospital, Suzhou,China(Longmei Bai, Wei Zhu);

17.Department of Neurology, Suzhou New District People’s Hospital, Suzhou, China(Runping Zhang, Jianping Li);

18.Department of Neurology, Suzhou Wuzhong Hospital, Suzhou, China(Qianqian Ding, Jiangang Zhu);

19.Department of Neurology, Kunshan Hospital of Traditional Chinese Medicine, Suzhou, China(Liqin Sheng, Hairong Ma);

20.Department of Neurology, Zhangjiagang Hospital of Traditional Chinese Medicine, Nanjing University of Chinese Medicine, Suzhou, China(Yaming Sun, Jufen Qian);

21.Department of Neurology, Changshu Hospital of Traditional Chinese Medicine, Suzhou, China(Juping Chen, Jianxing Yao);

22.Department of Neurology, Traditional Medicine Hospital of Taicang, Suzhou, China(Dan Xu, Lei Yuan);

23.Department of Neurology, Zhangjiagang Ao Yang Hospital, Suzhou, China(Liping Chen, Lihua Hu);

24.Department of Neurology, Kunshan Jen Ching Memorial Hospital, Suzhou, China(Qiang Zhang, Xu Chen).
